# Neuroendocrine Neoplasms of the Lungs, Thyroid, and Thymus

**DOI:** 10.3390/biomedicines13051028

**Published:** 2025-04-24

**Authors:** Barbara Buchalska, Małgorzata Solnik, Karol Maciejewski, Marta Fudalej, Andrzej Deptała, Anna Badowska-Kozakiewicz

**Affiliations:** 1 Students’ Scientific Organization of Cancer Cell Biology, Department of Oncology Propaedeutics, Medical University of Warsaw, 01-445 Warsaw, Poland; buchalskabarbara@gmail.com (B.B.); m.solnik98@gmail.com (M.S.); karol.maciejewski1998@gmail.com (K.M.); 2 Department Oncology Propaedeutics, Medical University of Warsaw, 02-091 Warsaw, Poland; mmfudalej@gmail.com (M.F.); andrzej.deptala@wum.edu.pl (A.D.)

**Keywords:** neuroendocrine tumour, lung, thyroid, thymus

## Abstract

Neuroendocrine neoplasms (NENs) comprise a group of tumours that can develop in various internal organs, but in this review, we will describe only those arising in the lungs, thyroid, and thymus. Pulmonary neuroendocrine neoplasms (pulmonary NENs) account for approximately 25% of all lung cancers. They are classified into four groups of tumours: typical carcinoids (TCs), atypical carcinoids (ACs), small cell lung carcinoma, and large cell lung carcinoma. This review focuses on TC and AC. The treatment consists mainly of radiotherapy, chemotherapy, and surgical resection, but novel drugs like atezolizumab are also utilised. The most common neuroendocrine neoplasm of the thyroid gland is medullary thyroid carcinoma (MTC), which commonly possesses *RET* protooncogene mutations. MTC is treated by a total thyroidectomy. Recently, tyrosine kinase inhibitors (TKIs) have emerged as an effective treatment option for patients with advanced MTC. Neuroendocrine tumours of the thymus (NETTs) are also being treated with a radical surgery.

## 1. Introduction

Neuroendocrine neoplasms (NENs) are a heterogenous group of malignancies originating from cells dispersed throughout the body [1]. The neuroendocrine cells originate from the neural crest [2]. They are characterised by two properties. The “neuro” aspect refers to the presence of dense core granules, similar to those in serotonergic neurons, which store monoamines [3]. The “endocrine” property means that they are able to synthesise and secrete those monoamines [3]. Their incidence is around 5.86/100,000 per year [4]. Neuroendocrine neoplasms are on the rise, and a 6.4-fold increase in incidence between 1973 and 2012 has been noticed, where it is thought to be caused by a growing awareness of NENs and improved medical imaging along with endoscopy [5]. NENs most commonly arise in the gastroenteropancreatic tract and lungs [6]. However, they can be found also in the respiratory tract, larynx, thyroid, breast, urogenital system, central nervous system, and skin [3]. They can be well, moderately, or poorly differentiated and have variable metastatic potential [7]. Furthermore, they can be functional or non-functional and if they give symptoms, they can reduce the quality of the patient’s life and therefore require therapy [7]. Given the histopathological complexity of NENs, the histological classification of those tumours has been a subject of intense debates, especially since the correct distinction of the types of NENs is crucial to the assignment of correcting treatment regimens. In this review, we present the current status of the World Health Organization classification of pulmonary, thyroid, and thymic NENs, as well as an overview of their clinical presentation, diagnosis, and treatment. Due to the distinct biology, clinical course, prognosis and therapeutic differences, aggressive neuroendocrine neoplasms such as small cell carcinoma and large cell carcinoma will not be discussed in this article.

## 2. Pulmonary Neuroendocrine Neoplasms

### 2.1. Epidemiology

Typical carcinoids (TCs) and atypical carcinoids (ACs) represent 1% of all lung cancers [8]. Their incidence in Europe and the United States is quite similar and ranges from 0.2 to 2 per 100,000 population/year [9]. Usually, they appear between the fourth and sixth life decade, although they are also the most common lung malignancy among children [9]. TC tends to occur in younger patients compared to AC [9]. The prevalence in women and men is relatively equal with a slightly higher incidence among women [9]. TC usually does not correlate with cigarette smoking [9]. On other hand, the prevalence of smokers within the AC patient group is twice as high as the general population (HR = 1.98, CI 0.63–6.19) [10,11]. Even though carcinoids occur mostly sporadic, around 5% of multiple endocrine neoplasia 1 syndrome (MEN-1) patients develop TC or less often AC [9]. Patients with MEN-1 typically develop tumours originating from the parathyroids, gastropancreatic tract, pituitary, foregut, adrenal glands, skin, and central nervous system [12].

### 2.2. Classification

The 2022 WHO Classification of Neuroendocrine Neoplasms divides them into four groups: TC, AC, small cell lung carcinoma (SCLC), and large cell lung neuroendocrine carcinoma (LCNEC). Although all of them are malignant, they differentiate in grading. SCLC and LCNEC are both high grade and TC and AC have a low and intermediate grade, respectively [13]. TC is diagnosed based on a mitotic rate of less than 2 mitoses per 2 mm^2^ in 10 high-powered fields and the absence of foci of necrosis. To compare, AC demonstrates from 2 to 10 mitoses and can contain necrosis (see Table 1). Although the Ki-67 labelling index is not an official diagnostic criterion, for TC it is usually lower than 5%, whereas for AC it is higher, but still less than 20% [13,14]. Based on the Ki-67 value, pulmonary carcinoids can be divided into three grades—G1 (TC with Ki-67 < 5%), G2 (TC with Ki-67 ≥ 5%), and G3 (AC) [15]. In TC with Ki-67 greater than or equal to 5%, the prognosis is worse, but better than in AC [15]. SCLC is divided into two subtypes: pure SCLC and combined SCLC. It is determined by the presence of features of a different histological carcinoma, non-small cell lung carcinoma (NSCLC), a variant, or one that contains at least 10% of a large cell carcinoma component. The mitotic rate, by definition, is greater than 10 per 10 high-power fields; nevertheless, the average mitotic rate is 80 mitoses per 2 mm^2^. Moreover, NSCLC is characterised by extensive intratumoural necrosis [16]. The criteria for microscopic diagnosis of LCNEC include numerous cell mitotic figures (>10/2 mm^2^, median 70/2 mm^2^), areas of necrosis, often extensive, sometimes with calcifications or in the central part of tumour nests, and high proliferative index (Ki-67 > 30%, most often 40–80%) [17].

AC and TC frequently have mutations in chromatin-remodelling genes. The most commonly mutated genes include *SWI/SNF* (SWItch/Sucrose Non-Fermentable), *MEN-1* (Multiple Endocrine Neoplasia type 1), *PSIP1* (PC4 And SRSF1 Interacting Protein 1), and *ARID1A* (AT-Rich Interaction Domain 1A) [18]. In contrast with SCLC and LCNEC, AC and TC rarely have mutations in *TP53* and *RB1* genes [18].

### 2.3. Clinical Presentation

Carcinoid tumours are mostly singular lesions located centrally (within mainstem, intermediate, lobar, or segmental bronchi) or less commonly on peripheral sites (within subsegmental bronchi or parenchyma) [19]. The last ones are usually asymptomatic and are found accidentally on imaging performed for different reasons. Central forms can present with respiratory symptoms including cough, chest pain, haemoptysis, dyspnoea, or recurrent chest infections [9]. The carcinoid syndrome is relatively rare and appears in approximately 5% of patients with AC and TC, although some of its symptoms such as flushing or diarrhoea may occur in up to 10% of patients [20].

### 2.4. Diagnostic Work-Up

TCs and ACs show a large amount of cytomorphological similarities. These lesions consist of monotonous, small in size cells with fine, or coarse granular chromatin patterns [10]. The chromatin is described as salt and pepper and no prominent nucleoli are present. Sometimes calcification, stromal amyloid, or stromal hyalinisation can be found [19]. The tumour cells are organised in palisading, organoid nesting, rosettes, and trabeculae. What differentiates AC from TC is more extensive cytologic atypia, and tumour necrosis presenting itself with focal patches and containing less than 10% of lesions volume [10]. For morphologic assessment, immunohistochemistry is used. Carcinoid tumours are positive for cytokeratin as well as neuroendocrine markers such as synaptophysin, chromogranin, and CD56. If the origin of the tumour is unknown, CDX2, PAX8, and TTF1 are performed [19].

TC and AC are commonly incidentally found on chest X-rays [9]. However, a standard imaging modality for diagnosis of the primary tumour is the contrast CT scan [21]. For staging and detecting distant metastases, multiphase CT or diffusion-weighted MRI may be used. Whole-body somatostatin receptor scintigraphy and ^18^Ga-DOTATATE PET scan can be useful to visualise the primary tumour, as well as to determine the TNM stage [9]. ^18^Ga-DOTATATE PET has a very high affinity to the SSR2 (somatostatin receptor 2) allowing for a detection of small lesions [22]. The sensitivity of ^18^Ga-DOTATATE PET in diagnosing lung carcinoids is 90% and the specificity—100% [23]. ^18^F-FDG PET can be performed; however, most of the TC show low or no uptake of ^18^F-FDG. Bronchoscopy enables biopsy in centrally located tumours, while transthoracic CT-guided biopsy is preferred for peripheral lesions. Nevertheless, with small biopsies it is difficult to distinguish TC from AC [9,24]. This is due to difficulties in assessing the mitotic index and the presence of necrosis in small biopsy specimens [25].

### 2.5. Treatment

In localised pulmonary carcinoids, surgical resection is the primary treatment option, yielding 5-year survival rates of 90% and 70% for TC and AC, respectively [26]. The primary objective of the surgery is to achieve a microscopically tumour-free resection margin (R0). The standard resection is an anatomical resection, typically involving lobectomy, bilobectomy, or pneumonectomy, depending on the location and size of the tumour. In the small peripheral TC (without nodal involvement and less than 2 cm), a sublobar resection (wedge or segmentectomy) may be performed. Peripheral AC should always be managed with lobar R0 resection, independent of tumour size, due to its higher malignant potential. In central tumours, the surgical objective is parenchyma-sparing surgery; thus, lung-sparing resections, such as bronchial sleeve resection and sleeve lobectomy, are preferred [27]. Radiotherapy or radiofrequency ablation are treatment options for patients with an inoperable peripheral tumour [28]. In advanced carcinoid cases, systemic therapy is employed, but surgical resection may also be considered for treating metastases, particularly those in the liver. This procedure can help control carcinoid syndrome cases as well as increase the 5-year overall survival (OS) rate up to 70% [9]. Watchful waiting can be considered in patients with diffuse idiopathic pulmonary neuroendocrine cell hyperplasia (DIPNECH) and cT1N0 carcinoids, in patients with MEN-1 syndrome, and patients with comorbid conditions [21]. There are limited randomised data on how to sequence available systemic therapies for pulmonary NENs. Treatment decisions are complex and must consider several aspects of a patient’s presentation, including histologic features, tumour growth rate, degree of symptoms, and hormone hypersecretion, as well as any concerning molecular or radiographic features [29]. In systemic therapy, one of the most utilised options are somatostatin analogues (SSAs). Due to their anti-secretive function, they are used as the first-line option in functioning carcinoids. Both PROMID and CLARINET trials with the use of SSAs in patients with functionally active or inactive NETs created a justification for SSAs (octreotide or lanreotide) use in non-functioning pulmonary carcinoids, as well [30,31,32]. In the SPINET study, the authors reported a clinical benefit of SSA therapy in TCs [33]. Peptide receptor radionuclide therapy (PRRT) is a treatment option for patients with inoperable lesions or with developed metastatic disease. The radiopharmaceutical binds to the somatostatin receptor, which is often overexpressed in carcinoids, delivering the high dose of radiation directly to lesions cells [9,26,34]. The outcome of its use is usually rather disappointing as it results in an overall response rate below 30% for 5-fluorouracil, dacarbazine, doxorubicin, and temozolomide (TMZ) [9,28]. Some studies showed the moderate efficacy of the cisplatin and etoposide combination, and TMZ alone or with capecitabine are some of the most commonly applied in chemotherapy [26,34]. Another therapy option is everolimus, an inhibitor of the mammalian target of rapamycin, in which the activation is increased in pulmonary NENs. In RADIANT-4 trial, the everolimus treatment showed increased progression-free survival (PFS) compared to placebo in patients with lung NENs subgroup [34,35]. RADIANT-4 is a randomised, double-blind, placebo-controlled, phase three study, in which patients with advanced progressive, well-differentiated, non-functional lung or gastrointestinal neuroendocrine tumours were enrolled. The median PFS was 11.0 months in the everolimus treatment, compared to 3.9 months in the placebo arm. Everolimus reduced the risk of disease progression or death by 52%. Only 10% of the patients developed adverse effects of the treatment, which included stomatitis, diarrhoea, fatigue, infections, rash, as well as peripheral oedema [35]. Furthermore, the LUNA trial established everolimus and SSAs, as well as the combination of those two, as a treatment strategy for pulmonary carcinoids [36]. Other evaluated treatment modalities include antiangiogenetic agents [9,28,34] and the epidermal growth factor receptor inhibitor [9,28]. Currently, the use of atezolizumab and bevacizumab in patients with TC and AC, in a phase II, single-arm, open-label study is being evaluated [37]. Bevacizumab is a monoclonal antibody, which targets vascular endothelial growth factor A (VEGF-A), thus inhibiting angiogenesis [38]. Atezolizumab, on the other hand, is a humanised immunoglobulin G1 monoclonal antibody, which binds to PD-L1 (programmed cell death 1 ligand 1). In a phase II study of atezolizumab monotherapy, the non-progression rate (NPR) in patients with lung NETs was 41.7% [39].

## 3. Thyroid Neuroendocrine Tumours

### 3.1. Epidemiology and Classificatiom

The most common neuroendocrine tumour of the thyroid gland is medullary thyroid carcinoma (MTC), and its incidence is 0.57/100,000 person-years (1–2% of all thyroid cancers) [40,41]. MTC originates from the parafollicular C cells of the thyroid gland, which become cancerous due to a mutation in the *RET* protooncogene [42]. The *RET* protooncogene encodes a receptor tyrosine kinase (RTK), which is required for the development of the kidneys, nervous system, and neuroendocrine cells as in the thyroid and adrenal gland [43]. The RET protein has a large extracellular domain with cysteine-rich and cadherin homology regions. The ligands of RET include the glial cell line-derived neurotrophic factor (GDNF) family of ligands (GFLs). These comprise GDNF, neurturin, artemin, and persephin [43]. The C cells secrete the peptide hormone calcitonin and calcitonin is a screening marker for occult MTC detection [42]. Specifically, the calcitonin concentration above 500 pg/mL is an indication for further imaging studies [44]. The 2022 WHO Classification introduced a grading scheme for MTC [45]. High-grade tumours have at least one of the following features—the presence of necrosis, mitotic count ≥5 per 2 mm^2^, and/or Ki-67 index ≥5%. The prevalence of high-grade tumours equals 25%. The tumour necrosis might be focal; therefore, using material from the biopsy is not recommended. The rest of the tumours are low-grade [45]. High-grade tumours have a worse prognosis—decreased OS, relapse-free survival, distant metastasis-free survival, and faster calcitonin doubling times [46].

### 3.2. Clinical Presentation

The most common clinical appearance of MTC is a single thyroid nodule. It may be accompanied by neck lymph nodes enlargement. The minority of patients may have systemic manifestations like diarrhoea, flushing, and Cushing’s disease due to the ectopic secretion of hormones [47].

### 3.3. Histopathology

MTC is a poorly delineated and infiltrative tumour, which may contain amyloid. Within its fibrous stroma, solid nests of discohesive cells are present [44]. The cells are polygonal with granular amphophilic cytoplasm and fine chromatin. The tumour shows foci of necrosis, calcification, ossification, haemorrhage, and a positive stain for mucin [48].

MTC is inherited as an autosomal dominant trait in 25% of patients, while 75% have a sporadic MTC. Together with a hereditary MTC, the multiple endocrine neoplasia type 2 (MEN-2) syndrome can occur. In MEN-2, apart from MTC, other tumours such as phaeochromocytoma and parathyroid adenomas are present [49]. The MEN-2 syndrome can be divided into MEN-2A and MEN-2B syndromes. MEN-2A is more prevalent—it affects 1/25,000 people. This condition is associated with MTC in 95% of the patients, pheochromocytomas in 50%, and hyperparathyroidism in 20–30%. The most lethal component of MEN-2A is MTC. On the other hand, hyperparathyroidism does not usually give any symptoms [50]. Patients with MEN-2A may also have cutaneous lichen amyloidosis and Hirschsprung’s disease [51]. The MEN-2B syndrome has a prevalence of 0.2/100,000. It is characterised by the presence of an aggressive and early-onset MTC. Patients also develop paragangliomas (50%), mucosal neuromas of the tongue, lips, eyelids, Marfanoid habitus, kyphoscoliosis, joint laxity, and ganglioneuromas of the gastrointestinal tract [50]. In sporadic MTC, point mutations of the *RET* gene are the most frequent, and the most common mutation is Met918Thr in exon 16. In *RET*-negative MTC, point mutations in the RAS gene can occur [49]. Some of the microRNA particles may also function as carcinogens or tumour suppressors in MTC. Among them, hsa-miR-375, hsa-miR-127-3p, and hsa-miR-429 are upregulated, and hsa-miR-199a-3p as well as hsa-miR-199b-5p are downregulated. The TGFB1 gene product, a member of the TGF-β superfamily, regulates hsa-miR-429 and hsa-miR-199a-3p. These microRNAs or TGFB1 gene can be a target of novel anti-cancer therapies [52].

MTC cells display very frequently on their surface the calcitonin receptor (CTR). CTR expression is associated with the expression of the cytoplasmatic phosphatase, tensin homolog deleted on chromosome 10 and osteopontin, along with the wild-type *RET/RAS* genes and the absence of tumour stroma. It indicates that the high CTR protein expression does not correlate with an aggressive MTC, but it occurs in more differentiated MTC cells [53]. Thyroid cancers show the expression of glucose metabolism-related proteins. MTC cells have higher levels of carbonic anhydrase 9 (CAIX) and CAIX activation supports the RET-mediated activation of the HIF pathway [54]. In 2020, it was shown that a novel biomarker, progastrin-releasing peptide (ProGRP) can be used in the differential diagnosis between MTC and non-MTC thyroid tumours. Patients with MTC have significantly higher levels of ProGRP concentration in serum compared to other patients with other thyroid nodules [55]. In the post-treatment follow-up of patients with MTC, procalcitonin (ProCT) can be also used as a marker. ProCT is a very stable protein, and it has a concentration-independent in vivo half-life of 20–24 h [56]. Carcinoembryonic antigen (CEA) may be elevated in patients with MTC [57].

Calcitonin-negative MTC is extremely rare, and it occurs without the elevation of the serum calcitonin [58]. This tumour can be diagnosed through the identification of high CgA serum level. At immunochemistry, calcitonin-negative MTCs present diffuse or focal positivity for calcitonin and CEA [58].

Another type of thyroid neuroendocrine tumour is thyroid paraganglioma. Thyroid paragangliomas are rare tumours that arise from the inferior laryngeal paraganglia. Patients with thyroid paraganglioma have normal calcitonin and CEA levels [59]. Thyroid paragangliomas are rarely malignant and do not secrete large amounts of catecholamines [59]. For differentiation between MTC and thyroid paraganglioma, immunostaining should be performed [60]. MTC is positive for calcitonin, CEA, and cytokeratins [60]. Thyroid paraganglioma stains positively for neuroendocrine markers but negatively for cytokeratins [60].

### 3.4. Treatment

The total thyroidectomy is a standard treatment for patients with MTC [61]. Serum calcitonin is a useful marker for MTC as a postoperative increase in serum calcitonin is associated with a recurrence [62]. Lymph node metastases are found in 75% of patients with MTC and the number of lymph nodes with metastases increases with an increasing concentration of CEA in serum. However, most patients with MTC and regional lymph node metastases are not cured by the total thyroidectomy because they have a systemic disease [63]. However, the prophylactic central, lateral, or contralateral neck dissections are recommended [64]. Systemic therapy for MTC includes treatment with tyrosine kinase inhibitors (TKIs) [65]. TKIs target proliferation-related pathways at various sites [66]. These drugs are being extensively used in the treatment of advanced MTC [66]. TKIs can be divided into multikinase inhibitors (cabozantinib, vandetanib), and selective inhibitors (pralsetinib, selpercatinib) [67]. Vandetanib comprises the most widely used TKI for advanced MTC. This drug was approved by the Food and Drug Administration (FDA) and European Medical Agency for the treatment of symptomatic, unresectable, locally advanced or metastatic MTC. It targets RET, vascular endothelial growth factor receptor (VEGFR), and epidermal growth factor receptor (EGFR) [68]. In 2024, Pitoia et al. reported that patients with MTC have a median reduction in the tumour diameter of 24.5% after a therapy with vandetanib or selpercatinib, proving its high efficacy [69]. Selpercatinib is a highly selective inhibitor of RET [70]. In a case series by Baek et al. (2023), it was shown that selpercatinib is effective in the treatment of MTC [70]. All studied patients had partial responses after treatment with this drug [70]. Cabozantinib, another FDA-approved drug, increases PFS in patients with progressive metastatic MTC. It targets the hepatocyte growth factor receptor (MET), VEGFR2, and RET [71]. In a study of MTC patients, Koehler et al. (2020) reported that OS and PFS are longer after a treatment with vandetanib than with cabozatinib (for OS 53 months vs. 24 months, for PFS 17 months vs. 4 months, respectively) [72]. An open-label, phase 1/2 ARROW study showed that the treatment with pralsetinib, a selective RET kinase inhibitor, resulted in complete response in 6.5% and partial response in 71.0% of the *RET*-mutant MTC patients [73]. Unfortunately, the treatment with TKIs is associated with various adverse effects, with hypertension being the most important [67]. Other side effects include diarrhoea, constipation, nausea, decreased weight, fatigue, rash, dry mouth, headache, musculoskeletal pain, and palmar-plantar erythrodysesthesia among others [67]. In 2020, a novel radionuclide therapy for MTC was introduced with a cholecystokinin 2 receptor agonist ^177^Lu-PP-F11N. ^177^Lu-PP-F11N is a radiolabelled minigastrin analogue that accumulates specifically in MTC cells and enables an effective biodistribution of the anti-cancer drugs [74].

Thyroid paragangliomas are treated with radical surgery [75].

## 4. Neuroendocrine Tumours of the Thymus

### 4.1. Epidemiology

Neuroendocrine tumours of the thymus (NETTs) are extremely rare [76]. To date, there are around 400 cases reported in the literature. The incidence is estimated at 0.18 per 1,000,000 population with a male-to-female ratio of 3:1 [77,78]. They account for less than 2% of all mediastinal tumours and about 5% of thymic malignancies [76]. These tumours occur mostly in the fifth and sixth decade of life, with an average of 58 years [78]. Contrary to lung neuroendocrine tumours, NETTs are not associated with a history of heavy smoking [79].

### 4.2. Classification

Similar to pulmonary neuroendocrine tumours, NETTs are divided into four types: low-grade TC, intermediate-grade AC, high-grade small cell carcinoma (SCC), and LCNEC [13,80].

The criteria used to differentiate TC and AC are the mitotic count and the presence of necrosis. TC is characterised by a mitotic count of less than 2 mitoses per 2 mm^2^ and the absence of necrosis, while AC has a mitotic count of 2–10 per 2 mm^2^ and may show foci of necrosis [13,81]. LCNEC and SCC of the thymus are both high-grade tumours. By definition, their mitotic rate is above 10 mitoses per 2 mm^2^, although it is usually much higher. Necrosis, often extensive, is a common finding in both types [80,82].

The most commonly mutated genes in AC and TC are *MEN-1* and *CDKN2A* (cyclin-dependent kinase inhibitor 2A) [83]. In SCC and LCNEC, alterations in *RB1*, *TP53*, *PTEN* (phosphatase and tensin homolog), and *MYC* are more common [83].

### 4.3. Clinical Presentation

TC and AC account for 20% and 40–50% of NETTs, respectively. Around 25% of typical carcinoid cases are associated with MEN-1 [80]. Thymic carcinoids are often locally invasive; at the time of diagnosis, up to 50% of patients show metastases to mediastinal lymph nodes or invasion of adjacent tissues [84]. Typically, they are either asymptomatic or present symptoms such as cough, dyspnoea, and hoarseness. Approximately 30–50% of cases are complicated by the paraneoplastic syndrome with the most common one being Cushing’s disease (50% of NETTs). Hyponatremia, acromegaly, or carcinoid syndrome are much less likely [80,84]. Paraneoplastic syndromes in NETTs are caused by the secretion of hormones, such as adrenocorticotrophic hormone (ACTH) in Cushing’s syndrome, by tumour cells [85]. AT has a 5-year survival rate of 56–78%, and TC has a rate of 97–100% [86].

### 4.4. Diagnostic Work-Up

For TC and AC imaging, a CT scan of the chest is the recommended modality. Multiphase CT or MRI are used to detect distant metastases in the liver and bones and are more useful in this regard than octreotide scans. The latter can be used to assess somatostatin receptor status and consequently, the efficacy of SSA use in therapy [84]. The biopsy and histopathology assessment is necessary for carcinoid confirmation. The tumour cells show typical neuroendocrine morphology (uniformly small cells with salt-and-pepper chromatin, etc.) and significant expression of neuroendocrine markers such as chromogranin and synaptophysin. A rosette pattern and trabecular formation are also present. Besides the necrosis findings, the atypia of tumour cells in AC is more pronounced than in TC [80].

For imaging high-grade tumours, several modalities are used, including enhanced chest CT, ^111^In-octreotide scan, and FDG-PET [81,82].

### 4.5. Treatment

Due to very limited data, the optimal treatment for NETTs has not been established yet. The main and only curative therapeutic option for TC and AC is radical surgery including the dissection of peripheral lymph nodes. Although the recurrence rate is high, it varies between 17% and 83% [21,81]. In patients receiving palliative care, debulking of large tumours is performed to alleviate symptoms [84]. Postoperative mediastinal radiotherapy and chemotherapy with platin and etoposide might be considered to improve local control [76]. For advanced cases, therapy options include SSAs, chemotherapy with a combination of platin and etoposide, or temozolomide alone as it has fewer side effects, everolimus, and PRRT [76,81]. Overall, the 5-year survival rate in TC is slightly better than in AC and ranges between 50 and 100%, while in AC, it ranges between 20 and 80% [80].

## 5. Conclusions

NENs of the lungs, thyroid, and thymus comprise a heterogenous group of malignancies, but they also show some similarities like the presence of dense core granules in the cells (see Table 2). The imaging of these tumours includes MRI, CT, X-ray, and ^18^Ga-DOTATATE PET scan. The last one shows tumours with somatostatin receptors with high specificity and sensitivity. The first line of treatment for these tumours is radical surgery. The adjuvant therapy includes SSAs and chemotherapy in pulmonary carcinoids, TKIs in MTC, and chemotherapy in NETTs. Recently, several clinical trials have been introduced to evaluate novel treatment options for the systemic treatment of these neoplasms. These novel treatment regimens may be particularly beneficial for patients with advanced or metastatic disease.

## Figures and Tables

**Table 1:** Summary of features of pulmonary neuroendocrine neoplasms.

Feature	Typical Carcinoid	Atypical Carcinoid
Differentiation	High	Intermediate
Mitoses per 10 HPF	<2	≤10
Ki-67	<5%	<20%
Necrosis	Absent	Focal if any
Presence of paraneoplastic syndrome	Rarely	Rarely
Prognosis 5-year OS	Around 90%	Around 70%

**Table 2:** Summary of features of NETs of the lungs, thyroid gland, and thymus. AC—atypical carcinoid; CT—computed tomography; FDG—fluorodeoxyglucose; FDOPA—fluorodopa; LCNEC—large cell neuroendocrine carcinoma; MRI—magnetic resonance imaging; MTC—medullary thyroid carcinoma; NETs—neuroendocrine tumours; PET—positron emission tomography; PRRT—peptide receptor radionuclide therapy; SCC—squamous cell carcinoma; SCLC—small cell lung carcinoma; SSAs—somatostatin analogues; USG—ultrasonography.

	Pulmonary NETs	Thyroid NETs	NETTs
Types	TC, AC, SCLC, LCNEC [13]	MTC, thyroid paraganglioma [41,59]	TC, AC, SCLC, LCNEC [80]
Clinical presentation	- Centrally located tumours—Cough, chest pain, haemoptysis, dyspnoea, recurrent infections - Peripherally located tumours—often asymptomatic [9]	Single thyroid nodule [47]	Asymptomatic or cough, dyspnoea, hoarseness, paraneoplastic syndromes [80,84]
Imaging	X-ray, CT, MRI, Somatostatin Receptor Scintigraphy [9]	USG, CT, MRI, 18F-FDOPA PET/CT, 18F-FDG PET/CT, Somatostatin Receptor Scintigraphy [87]	CT, MRI, Somatostatin Receptor Scintigraphy [80]
Treatment	- Surgical resection—for localised tumours [26] - SSAs—for functioning tumours [31] - Radiotherapy, radiofrequency ablation, PRRT—for inoperable tumours [9,28] - Chemotherapy—5-fluorouracil, dacarbazine, doxorubicin, temozolomide [9] - Targeted drugs—everolimus, atezolizumab, bevacizumab [9,37]	- Surgical resection [61] - TKIs [65]	- Surgical resection [21,81] - Radiotherapy [76] - Chemotherapy—platin, etoposide, temozolomide [76] - PRRT [76] - SSAs [76] - Targeted drugs—everolimus [76]
